# The Impact of Roads on the Demography of Grizzly Bears in Alberta

**DOI:** 10.1371/journal.pone.0115535

**Published:** 2014-12-22

**Authors:** John Boulanger, Gordon B. Stenhouse

**Affiliations:** 1 Integrated Ecological Research, Nelson, British Columbia, Canada; 2 Foothills Research Institute, Hinton, Alberta, Canada; University of Lleida, Spain

## Abstract

One of the principal factors that have reduced grizzly bear populations has been the creation of human access into grizzly bear habitat by roads built for resource extraction. Past studies have documented mortality and distributional changes of bears relative to roads but none have attempted to estimate the direct demographic impact of roads in terms of both survival rates, reproductive rates, and the interaction of reproductive state of female bears with survival rate. We applied a combination of survival and reproductive models to estimate demographic parameters for threatened grizzly bear populations in Alberta. Instead of attempting to estimate mean trend we explored factors which caused biological and spatial variation in population trend. We found that sex and age class survival was related to road density with subadult bears being most vulnerable to road-based mortality. A multi-state reproduction model found that females accompanied by cubs of the year and/or yearling cubs had lower survival rates compared to females with two year olds or no cubs. A demographic model found strong spatial gradients in population trend based upon road density. Threshold road densities needed to ensure population stability were estimated to further refine targets for population recovery of grizzly bears in Alberta. Models that considered lowered survival of females with dependant offspring resulted in lower road density thresholds to ensure stable bear populations. Our results demonstrate likely spatial variation in population trend and provide an example how demographic analysis can be used to refine and direct conservation measures for threatened species.

## Introduction

One of the primary factors that has reduced grizzly bear populations in some portions of North America, has been the effects of unsustainable human caused mortality which has been linked to the creation of human access into prime bear habitat [1]–[3]. Roads have also affected movements and distribution of bears [4]–[6], changes in behavior relative to roads [7], and changes in body condition and survival rates relative to roads [8], and have caused fragmentation of populations [9].

The status designation for grizzly bears in the province of Alberta, Canada was changed to threatened in 2010 and a provincial recovery plan for this species has been in place since 2008. [10], [11]. With ongoing resource extraction activities and increasing levels of human use within grizzly bear habitat in Alberta [12], [13] there is a need to better monitor grizzly bear populations and determine quantitative thresholds for levels of anthropogenic disturbance that can potentially cause negative trends on both population size and recovery efforts.

Although the literature contains numerous publications looking at the negative consequences of roads for grizzly bear populations [2], [14]–[16] we were interested in estimating the direct demographic consequences of roads. We made no attempt to quantify or explore the level of human use on these roads, but rather focused on their presence and abundance on the landscape. Our focus was to determine whether survival and reproductive rates of grizzly bears were inter-related in the context of roads on the landscape due to the fact that other studies have documented differential distributions of females with cubs relative to roads. Of the 42,598 km of roads in our study area, 96.5% are resource-based two lane gravel roads. Other studies [4], [6], [7] have shown that bears select habitat in the proximity of these gravel resource-based roads and therefore the primary effects of the roads are more likely to be increased risk of human encounter and mortality [2] than fragmentation effects caused by inability to cross the roads [9].

Our primary objective was to estimate demographic parameters for collared grizzly bears monitored in Alberta from 1999–2012 and explore whether variation in these parameters could be related back to road densities and other anthropogenic factors. We believe it is as important to understand sources of variation in population trend as to estimate a single point estimate of trend for bear populations given issues with sparse geographical and temporal sampling coverage, and the reality of limited conservation resources with which to monitor a species which occurs in relatively low densities in remote habitats. We use survival analyses with covariates to estimate variation in survival based upon anthropogenic factors. These survival covariates are then linked to a demographic model to explore variation in population trend of grizzly bears, and provide management options to facilitate recovery based on the findings of our demographic model. Our analysis focused on the home range to watershed scale as opposed to larger-scale analysis of fragmentation effects caused by major highways [9]. We believe that this general methodology can be applied to focus conservation strategies for other species at risk and to guide both population recovery efforts and land use management decisions.

## Materials and Methods

### Study area

Our study area was divided into Alberta grizzly bear management units [9] where the topography varies from plains and foothills to subalpine and high alpine areas. The total estimated area of grizzly bear habitat in Alberta is 91,290 km^2^ of which 32% is in protected areas, 41% in core and 27% in secondary habitat zones [17] Previous research indicated that major highways fragmented populations of bears within each population unit [9]. Of 42,598 km of roads within potential grizzly bear habitat, 41,106 km (96.5%) are gravel secondary roads in association with settlements and a legacy of resource extraction industrial activities which continues today. These existing gravel road networks provide human access into grizzly bear habitat and are also used by the public for a variety of recreational activities throughout the year. A history of forest fires, forest harvesting, mining, and energy exploration and development has created a mosaic of forest types and stand ages, as indicated by regenerating forest habitats and an array of permanent road networks [18]–[21]. Our study area also included federal and provincial parks and protected areas such as Whitehorse Wildlands Park, and the Wilmore Wilderness Area where anthropogenic changes in habitat are uncommon and motorized road access features are low in number.

### Field methods

**Figure 1:**
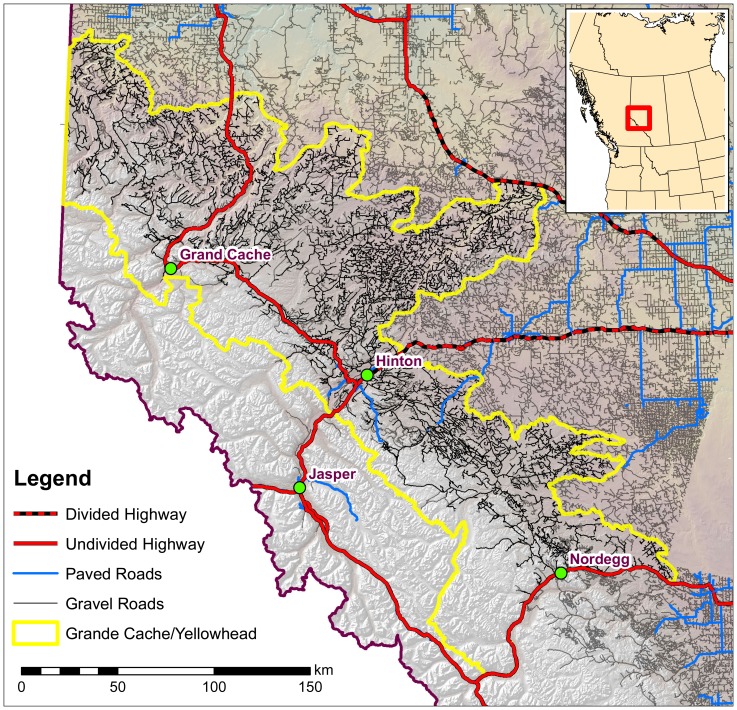
The focal study area for the Foothills Research Institute grizzly bear project in Alberta, Canada. The Grande Cache (north of Hinton) and Yellowhead (south of Hinton, AB) are displayed. The majority of collared bears in the analysis were within these 2 management units.

The data used for this analysis was collected from 1999–2012 as part of a larger study by the Foothills Research Institute Grizzly Bear Program which was mainly conducted in the Grande Cache and Yellowhead population units (Fig. 1). This larger study initially focused on remote sensing based habitat mapping [22] and collecting grizzly bear location data for the creation of regionally appropriate resource selection function mapping [21] for the period 1999–2005. After this time the research focus altered to monitoring individual bears in relation to anthropogenic landscape conditions and change in Alberta [12], [13] and bear health characteristics [8], [23]–[25]. Grizzly bears were captured and collared each spring (May-June) between 1999–2012 using either helicopter aerial darting, leg-hold snares, and culvert traps [26], [27]. Aerial captures typically occurred in subalpine habitats or in forestry cut-blocks where helicopter operations were possible. Most snare sites, on the other hand, were at lower elevations and normally within 100 m of a road. Some capture activities took place in the fall however the number of these events was limited. Beginning in 2006, capture efforts (2006–2010) were focused on the use of culvert traps and helicopter aerial darting, with the use of foot snares phased out [28]. Global Positioning Systems (GPS) radiocollars from Televilt Simplex, and Tellus (Lindesberg, Sweden) (1999–2010), and Advanced Telemetry Systems (ATS) (Isanti, Minnesota) (1999–2001) were fitted on captured bears and programmed to acquire a location every 1–4 hours. In addition, very high frequency (VHF) ear tag transmitters (ATS) were fitted on all captured bears. Standard morphological measurements were obtained from each bear along with biological samples (blood, hair, etc.) and a pre-molar tooth was extracted for ageing purposes [29]. All grizzly bears captured and handled during the course of this research program were authorized under the permitting authority of the Alberta Department of Environment and Sustainable Resource Development (provincial jurisdiction lands), Alberta Tourism and Parks (provincial parks and protected areas jurisdiction lands), and Parks Canada (federal jurisdiction lands). Research and collection permits were obtained each year of study from all these regulatory agencies. All capture efforts followed guidelines by the Canadian Council of Animal Care [30] and the American Society of Mammalogists [31], and were approved annually both by the University of Saskatchewan's Committee on Animal Care and Supply and the Alberta Department of Sustainable Resource Development Animal Care Committee. Data sets related to landscape conditions (roads, habitat changes) were prepared from remote sensing imagery updated annually to correspond with GPS collar location data sets for specific years [13], [18], [32].

### Survival analysis

#### Adult and subadult survival

Known fate binomial models in program MARK [33] were used to estimate survival for age and sex groups with adults and subadults being defined as bears that were 4 years of age or younger. In most years no bears were monitored during the denning season, approximately January to March. Therefore these months did not contribute to yearly survival rate estimate data. To account for this, the time interval for a month for MARK known fate models was set at one over the number of months in which bears were monitored (9) for a given year.

The influence of road density and age group on survival rates was considered in model selection efforts. Anthropogenic variables were defined from remote sensing-based land cover mapping and databases of anthropogenic footprints [18], [34]. The main anthropogenic covariates considered were road density (*roads*) which was the kilometers of all weather (gravel or paved) road encountered within a 300 m radius of each GPS location. This scale was chosen since it described the actual areas that bears traversed and the relative risk that they were directly exposed [35], [36]. In addition, the distance of each GPS bear location to roads (*droads*) or other anthropogenic features was considered as an alternative indicator of risk. The average of each covariate for the duration a bear was collared was then summarized as an individual covariate to provide a general index of anthropogenic factors that a bear encountered while traversing its habitat. Models were built sequentially to determine the most parsimonious descriptor of variation in age and sex-specific survival rates and how roads potentially influenced the survival rates of each age and sex class. Model fit was compared using the sample-size-corrected Akaike Information Criterion (AIC_c_) index of model fit [37]. The model with the lowest AIC_c_ score was considered the most parsimonious, thus optimizing the tradeoff between bias and precision [37]. The difference between any given model and the most supported (ΔAIC_c_) was used to evaluate the relative fit of models when their AIC_c_ scores were close. In general, any model with an ΔAIC_c_ score of ≤2 was supported by the data.

#### Cub survival

Estimation of cub-of-the-year survival is challenging given that these cubs are not radio collared and were not captured within our long term research program. Instead, repeated observations of females with cubs, usually taken from helicopters during downloads of GPS collars, are used to determine if cubs have survived. However seldom are cub mortality events actually seen, especially in a largely forested landscape, so the time of loss of cubs is uncertain. All that can usually be determined is that cubs were lost between the time that a female was observed with cubs and the time she was observed without a cub or any cubs. Nest survival models [38] were applied to estimate survival of cubs and yearlings. The inputs of the nest survival models were the number of days since April 1^st^ (earliest date of den emergence) when the dependent offspring were first observed (*k*), when the cubs were last seen alive (*l*) and the last day the female with cub(s) were seen (*m*), and from this information the fate of the cubs or yearlings. If cubs survived to the denning season, then *l* will equal *m*. If the cubs died, then *m* is the first day the female was seen without cub(s), and *l* is the last day the cub(s) were seen alive. It is assumed that mortality occurred between *l* and *m*. We calculated daily survival rates based on the interval between the first observations of cubs in the spring and the last observation of cubs in the fall. The daily survival rates were then converted to an estimate of survival for this period. Bootstrap methods with resampling conditioned on individual bear litters was used to estimate variances to account for non-independence of litter groups [39].

### Reproductive analysis

We estimated litter size from the initial observation of females with cubs after emergence from dens in the spring. If multiple observations were made, the observation with the maximum number of cubs observed was used to account for cases in which not all cubs were first observed. If observations occurred after emergence from dens it was possible that mortality prior to sightings reduced the number of cubs which would negatively bias litter size. We adjusted litter size by first estimating daily cub survival rate (from the cub survival analysis) and then estimating the survival rate of cubs during the mean interval from den emergence (assumed to be April 1^st^ each year) to the time an observation was made [39], [40]. We divided the mean number of cubs by the survival rate for the mean interval to arrive at an adjusted cub litter size.

We used a multi-state model [39], [41] to model and estimate reproductive rate for adult female grizzly bears. This approach subdivides yearly observations of adult females into females with cubs(C), females with yearlings (Y), females with 2 year olds (T), and females without cubs (N). It then estimates the probability of females transitioning between the biologically plausible states with transitions denoted as the previous then current state (NN, NC, CY, CN, CC, YC, YT, YN, TN, and TC). This approach efficiently confronts issues with the longer inter-birth interval of grizzly bears created by dependant offspring. Initially, models that estimated the 10 plausible transitions were used to estimate base reproductive rate. Non-sequential yearly detections were not considered in the analysis. Bears aged 3 and above were included in the analysis since a bear that had cubs at age 4 would become pregnant at age 3 (therefore providing age-specific data on the non-cub to cub transition probability).

Reproductive rate was estimated using transition probabilities from the base multi-state model. The transition probabilities were projected in a matrix model (described later) to estimate the stable state proportions of females with cubs, yearlings, two year olds and no cubs. The stable state proportion of females with cubs was then multiplied by the adjusted female litter size to estimate reproductive rate as the yearly number of female cubs per adult female in the population. Litter size was divided by 2 to obtain the number of female cubs under the assumption of an equal sex ratio at birth [39]. A parametric bootstrap method that conditioned on individual bears in the data set and repeated the above estimation procedure 100 times was used to estimate the variance of the reproductive rate.

This multi-state model estimate was also compared to the simpler reproductive estimator of McLellan [42] which is simply the total number of cubs produced divided by the number of bear years in which adult females were monitored. A bootstrap estimator [43] was also used to obtain a variance estimate for this ratio.

We investigate mortality risk associated with a female having dependant offspring compared to females without offspring by expanding the multi-state reproductive rate model to include an absorbing dead state. This “D” state was entered into the encounter matrix for the subsequent year when a female was killed. The transition probabilities from each reproductive state to the dead state were then estimated. The death transition probability in this context was defined as the probability that a female bear would be dead before the following den emergence year. In this context, the dead state analyses estimates the potential reduction of reproductive rate by the death of the female bears with dependant offspring (assuming that cubs and yearlings cannot survive if orphaned). Annual survival rate for each reproductive state was estimated as 1 minus the transition probability.

Of further interest was the relationship between age and road density on the reproductive and death state transitions. We therefore considered models that assessed age-specific transitions and the influence of road density on each of the transitions. Of key interest was whether females with dependant offspring displayed different survival rates than females without dependant offspring, and whether all females were equally vulnerable to human caused mortality as indexed by road covariates. As with the known fate analysis, model fit was assessed using AIC_c_ information criterion methods.

### Demographic model analysis

A demographic model was used to assess the effects of roads on overall population trend of grizzly bears. Of particular interest was determination of threshold values of road density where overall population trend was reduced to create sink habitat [17]. The base model used for this analysis was a stage-based matrix model [1], [40], [44]. This model only included female bears and was subdivided into cubs, yearling, two year old/subadult, and adult stages. Reproductive rate was modelled using the transition probabilities from the multi-state model analysis. A bear was considered an adult when it was first able to conceive which was at three years of age as defined in the multi-state model analysis. We assumed that, although there have been reports of yearlings surviving after their mother had been killed, in general both cubs of the year and yearling cubs would not survive the loss of their mother. Proportions of females at different reproductive states were initially set at stable distributions as determined by the multi-state reproductive analysis. The model was projected 100 years to allow assessment of stable state distributions of stage classes and asymptotic estimates of population trend (λ) [45].

The principal objective of this model was to assess sensitivity of lambda to road density as opposed to estimate overall population trend for the Alberta study area. This was done by modelling the effect of road density on survival rates. We felt that sparse coverage of radio collared bears across the landscape and likely temporal and spatial variation in survival, reproductive, and subsequent vital rates precluded the ability to robustly estimate a mean population trend [44], [46]. Instead, the model provided further assessment of likely sink and source areas of Alberta as a function of road densities. We defined sink habitat as a watershed unit where combinations of survival rates created estimates of lambda that were less than 1 under the assumption that emigration and immigration rates into watershed areas would be equal. We used our results to categorize grizzly bear watershed units (GBWU) in terms of road thresholds and associated population risk. Grizzly bear watershed units are based on major watersheds subdivided along heights of land and occasionally along watercourses, to approximate the size of an adult female grizzly bear home range (∼500 km^2^). [10], [11].

We obtained confidence limits for estimates of lambda from the demographic model by bootstrapping the component demographic model analyses (known fate, cub survival, and multi-state reproductive model). For each bootstrap run the original data sets were randomly resampled and estimates run through the respective program MARK models for 1000 iterations. Confidence limits were based upon the 2.5^th^ and 97.5^th^ percentiles of lambda estimates from the bootstrap procedure. The PopTools plug in [47] to Excel was used to cross check estimates of lambda and generate stable age distributions from the matrix model. Our estimates of lambda will contain both sampling and temporal variance [46], [48]. Sample sizes precluded estimation of process variance for each of the component life history parameters.

## Results

### Survival analysis

#### Adult and subadult survival

Although data sampling occurred throughout provincial grizzly bear management units (BMA's) the majority of bears were monitored in the Yellowhead (53 bears) and Grande Cache (53 bears) management units (Fig. 1) with 36 bears monitored in the other grizzly bear management areas. As an initial step, grizzly bear management units were entered into the analysis and there was minimal support for GBU-specific survival rates. Therefore, data was pooled across management units for the rest of the analyses. Sample sizes of bears (and deaths) were 51 (4) adult females, 40 (5) adult males, 24 (6) subadult females, and 27 (7) subadult males monitored monthly from 1999–2012. Of the deaths, 7 were illegal poaching, 3 were legal hunted (grizzly bear hunting was curtailed in Alberta in 2006), 4 were management actions, 2 were road kills, 1 was natural, and 5 were classified as unknown. Of the 22 mortalities, 19 were located less than 500 meters from a road with only 1 mortality in a wilderness area (distance from road = 9.7 km). Of the 19 mortalities associated with roads, only 1 was in the proximity (distance = 60 meters) of a paved primary road and the rest were closer to secondary resource roads (S1 Table and S1 Fig.). Because mortalities were located by radio collar signals this finding could not be attributed to increased visibility of bear mortalities near roads.

Model selection focused on determining the most parsimonious model that described variation in survival rates due to age, sex, and the exposure of bears to road density. A model with sex-specific survival rates and an interaction of road density with age was most supported (Model 1, Table 1) suggesting that each sex of bear had unique survival rates but survival of bears relative to roads was associated with bear age (not sex). This model was more supported than other sex, age, and roads models (models 2–8), models with distance to roads/anthropogenic features (Model 11) as well as models that considered each covariate separately (models 9, 13, and 15), or models with year-specific effects (Models 17 and 19).

**Table 1:** Known fate survival analysis model selection results of grizzly bears in Alberta, Canada.

No	Model	AIC_c_	ΔAIC*_c_*	*w_i_*	K	Deviance
1	sex+age*roads	189.7	0.00	0.260	4	181.6
2	sex+roads	190.4	0.75	0.179	3	184.4
3	sex+age+roads	191.3	1.65	0.114	4	183.3
4	sex+age+age*roads	191.6	1.95	0.098	4	183.6
5	sex*age+age*roads	191.9	2.29	0.083	6	179.9
6	sex+sex*roads	192.1	2.49	0.075	4	184.1
7	sex*age+roads	192.8	3.16	0.054	5	182.8
8	age+roads	193.3	3.69	0.041	3	187.3
9	roads	194.0	4.33	0.030	2	190.0
10	sex+age+sex*roads	194.6	4.98	0.022	6	182.6
11	droads	195.2	5.54	0.016	2	191.2
12	sex*age*roads	195.2	5.57	0.016	8	179.1
13	sex	198.4	8.78	0.003	2	194.4
14	sex+age	199.1	9.43	0.002	2	195.1
15	age	199.1	9.43	0.002	2	195.1
16	sex*age	199.1	9.43	0.002	4	191.1
17	sex*age+year	200.0	10.30	0.002	5	189.9
18	constant	202.1	12.44	0.001	1	200.1
19	year	203.2	13.50	0.000	2	199.2

The covariate roads described road density (km roads/km^2^) and the covariate droads described distance from roads or other anthropogenic features. Sample-size adjusted Akaike Information Criteria (AIC*_c_*), difference in AIC*_c_* between most supported and given model (ΔAIC*_c_*), Akaike weight (*w_i_*), the number of parameters (K), and Deviance are shown.

**Figure 2:**
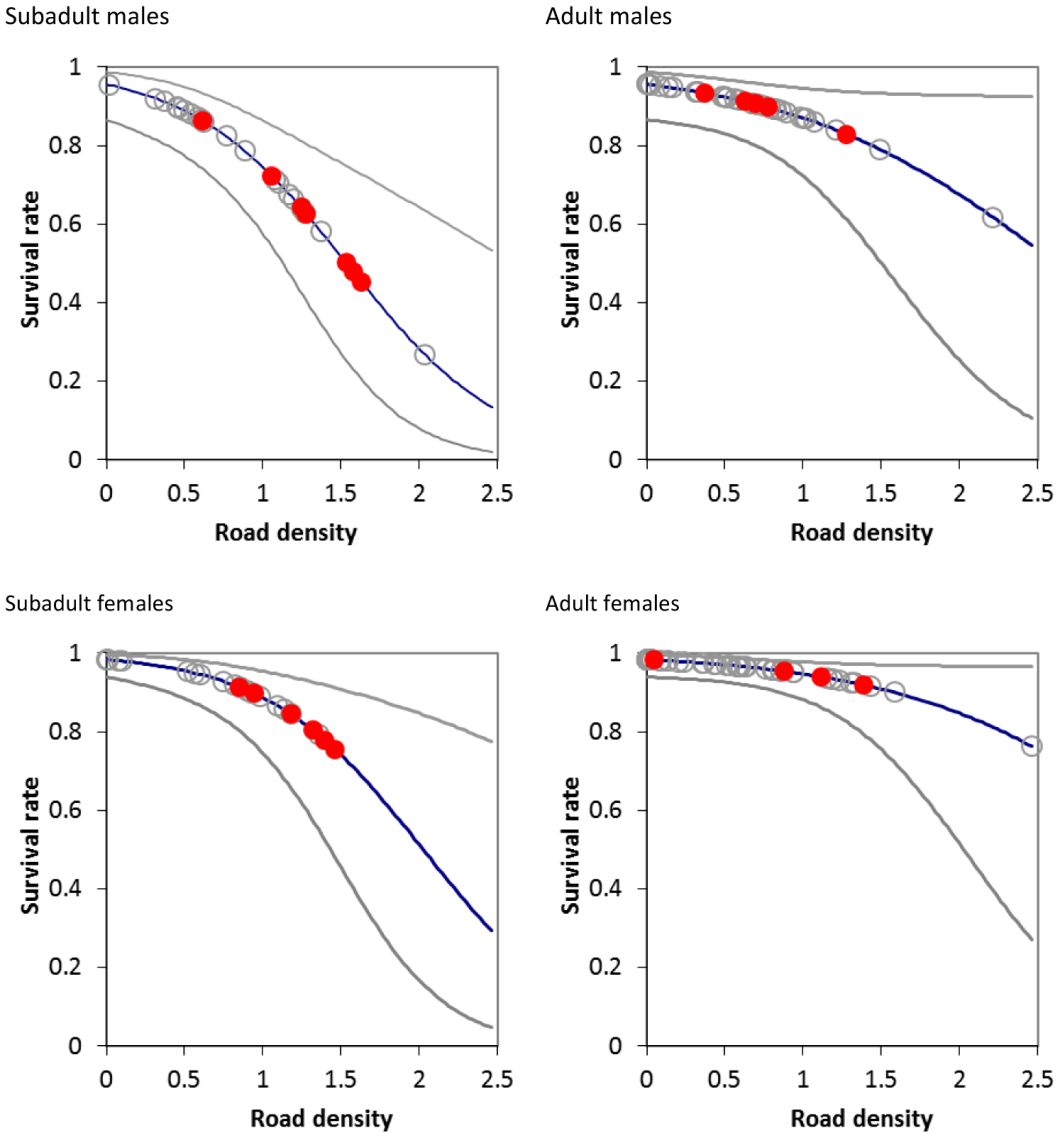
The estimated relationship between road density and age and sex class from known fate model analysis of Alberta grizzly bear data (Model 1, **Table 1**). Data points for individual bears are shown as circles with mortalities denoted as black dots. Grey lines indicate 95% confidence intervals for predictions.

Sex and age-specific survival rate estimates (Table 1, Model 16) showed higher survival rates for female bears compared to male bears, and adults compared to subadults (Table 2). Plots of age and sex specific survival rates revealed that subadult bears show a larger reduction in survival rates relative to roads compared to adult bears with the largest reduction for subadult males (Fig. 2). The distribution of collared bears relative to road density shows most bears occurred within road densities of 1.5 km/km^2^ or less with most mortalities occurring at road densities of greater than 1 except for adult males where mortalities occurred across all road densities.

**Table 2:** Model averaged estimates of survival from known fate models (Table 1) of grizzly bears in Alberta, Canada.

Sex	Age	Estimate	Std. Error	Confidence limit	
Male	Sub adult	0.78	0.08	0.59	0.90
	adult	0.88	0.05	0.75	0.95
Female	Sub adult	0.88	0.06	0.70	0.96
	adult	0.96	0.02	0.90	0.99

Estimates were based on mean covariate values.

#### Cub survival

Records from twenty four adult females with a total of 53 cubs were used for the cub survival analysis. Of the 53 cubs, there were 17 documented mortalities. This summary included one case of a female with 2 cubs being killed therefore causing death of the cubs. The maximum interval from first to last observation of cubs was 210 days with daily survival rate of 0.9971555 (SE = 0.00069, CI = 0.9995426–0.998232) which resulted in a survival rate estimate of 0.55 (SE = 0.09, CI = 0.35–0.74) for the entire 210 day monitoring period.

### Reproductive analysis

#### Estimation of reproductive rate

Fifty four adult female bears were used in the reproductive state analysis with 196 bear-year observations of reproductive state. Of the 54 bears, 9 were mortalities of which 6 were human caused (2 deaths were unknown for females without cubs). One female that was a mortality was not monitored for reproductive state on the year it was killed (S2 Table) The average number of years a female bear was monitored was 3.29 (Std. Dev. = 1.62, Min = 2. Max = 9, n = 54).

Estimates of transition to the dead state suggested higher chances of mortality for females with cubs and yearlings compared to females with no cubs and two year olds (Table 3). For example, of 16 bears that had yearlings, two ended up dead by the spring of the following year which resulted in a transition estimate of 0.17. In comparison, of the 60 bears that did not have cubs, only 3 ended up as mortalities in the following year. The net result was that reproductive transitions, especially transitions from cubs to yearlings and yearlings to two year olds were reduced when a dead state was included when the mortality risk was included in the analysis.

**Table 3:** Summary of sample sizes of events (*n*) and transition probabilities (*p*) from multi-state model reproductive analysis of grizzly bears in Alberta, Canada.

Current	Transfer to:									
	No cubs		Cubs		Yearlings		Two year olds		Dead	
	n	p	n	p	n	P	n	p	n	p
Dead state										
No cubs	29	0.533	28	0.425					3	0.042
Cubs	1	0.044	2	0.089	17	0.795			2	0.073
Yearlings	3	0.285	2	0.018			7	0.529	2	0.168
Two year olds	7	0.607	3	0.364						0.029
No dead state										
No cubs	29	0.579	28	0.421						
Cubs	1	0.090	2	0.118	17	0.802				
Yearlings	3	0.283	2	0.068			7	0.649		
Two year olds	7	0.794	3	0.206						

Estimates were from a constant parameter multi-state model with and without a dead state.

The transition probabilities from the multi-state analysis (without the dead state) were projected in a matrix model to estimate the stable state proportions of bears in each of the reproductive classes. From this, it was estimated that 25.8% of female bears were with cubs each year and 20.7%, 11.9%, and 41.7% of bears were with yearlings, two year olds, or without cubs in a given year.

The unadjusted mean number of cubs per female observed was 1.79 (total cubs observed = 77, n = 43, std. = 0.69, min = 1, max = 3). The mean duration of days between April 1^st^ and first observation of cubs was 57.2 days. Using an estimate of cub survival of 0.55 (SE = 0.09, CI = 0.35–0.74) with a resulting daily survival rate estimate of 0.9971555, the number of cubs produced of was estimated to be 90.63 which resulted in an adjusted mean number of cubs of 2.11 per female.

The multi-strata based reproductive rate estimate [39] was the stable state proportion of females with cubs (25.8%) times the average number of female cubs per female bear (2.11*0.5) which resulted in a reproductive rate of the Alberta bears of 0.272 (2.11*0.258*0.5) female cubs per female per year (SE = 0.047, CI = 0.20–0.41). In comparison, the estimate of reproductive rate from [42] was the adjusted total number of cubs (85.2) divided by the number of bear years that females four years old or greater were monitored (184 bear years). The resulting estimate was (90.7/184*0.5) or 0.246 female cubs per female per year (SE = 0.034, CI = 0.16–0.29).

#### Factors influencing reproduction

Model selection focused initially on the development of a parsimonious base model to allow further exploration of factors influence age specific reproduction and mortality risk. From this a model that pooled the transitions of reproductive states (with offspring) back to cubs was supported. In addition, pooling mortality risk of females with no cubs/two year olds and cub/yearling was supported (Table 4, Model 12). Age specific reproduction was assessed next with age-specific models for all transitions considered as well age-specific mortality risk. A model with age-specific transition from NC and age-specific mortality risk was most supported (Table 4, Model 6). Finally, influence of roads on mortality risk for various reproductive states was considered with a focus on whether females with cubs of the year or yearlings were at higher mortality risk with increasing road density compared to other classes. This analysis suggested that all classes were equally vulnerable to the mortality influence associated with roads, however females with cubs or yearlings had higher mortality risk compared to females with two year olds or no cubs (Table 4, Model 1).

**Table 4:** Model selection for multi-state reproductive model analysis of grizzly bears in Alberta, Canada.

No	Reproduction	Survival	AICc	ΔAICc	w_i_	K	Deviance
*Survival covariates*							
1	NC(age) CY YT	(NT CY) +roads	559.6	0.00	0.327	10	538.0
2	NC(age) CY YT	(NT CY) +roads+age	560.2	0.61	0.241	11	536.3
3	NC(age) CY YT	NT CY +roads	561.1	1.51	0.154	10	539.6
4	NC(age) CY YT	NT +roads CY+roads	561.8	2.20	0.109	11	537.9
5	NC(age) CY YT	NT C+roads Y+roads	563.4	3.80	0.049	11	539.5
*Age-specific reproduction* A							
6	NC(age) CY YT	(NT CY) +age	563.3	3.68	0.052	10	541.7
7	NC(age) CY(age) YT	(NT CY) +age	565.2	5.63	0.020	11	541.3
8	NC(age^2^) CY YT	(NT CY) +age	565.4	5.82	0.018	10	543.9
9	NC(age) CY YT	(NT CY)	565.9	6.30	0.014	9	546.6
10	NC(age) CY(age) YT	NT+age CY+age	567.2	7.58	0.007	12	540.9
11	NC(age) CY(age) YT(age)	(NT CY) +age	567.6	7.98	0.006	12	541.3
*Base models*							
12	NC CY YT CC = YC = TC	NT CY	570.7	11.04	0.001	8	553.6
13	NC CY YT CC = YC = TC	NT T Y	571.2	11.63	0.001	9	552.0
14	NC CY YT CC = YC = TC	C Y NT	571.8	12.20	0.001	9	552.5
15	NC CY YT CC = TC YC	NT CY	572.8	13.15	0.000	11	548.9
16	NC CY YT CC TC YC	N C Y T	572.8	13.22	0.000	9	553.6

Sample-size adjusted Akaike Information Criteria (AIC*_c_*), difference in AIC*_c_* between most supported and given model (ΔAIC*_c_*), Akaike weight (*w_i_*), the number of parameters (K), and Deviance are shown.

A A model with CC = YC = TC was used for all models in this group.

**Figure 3:**
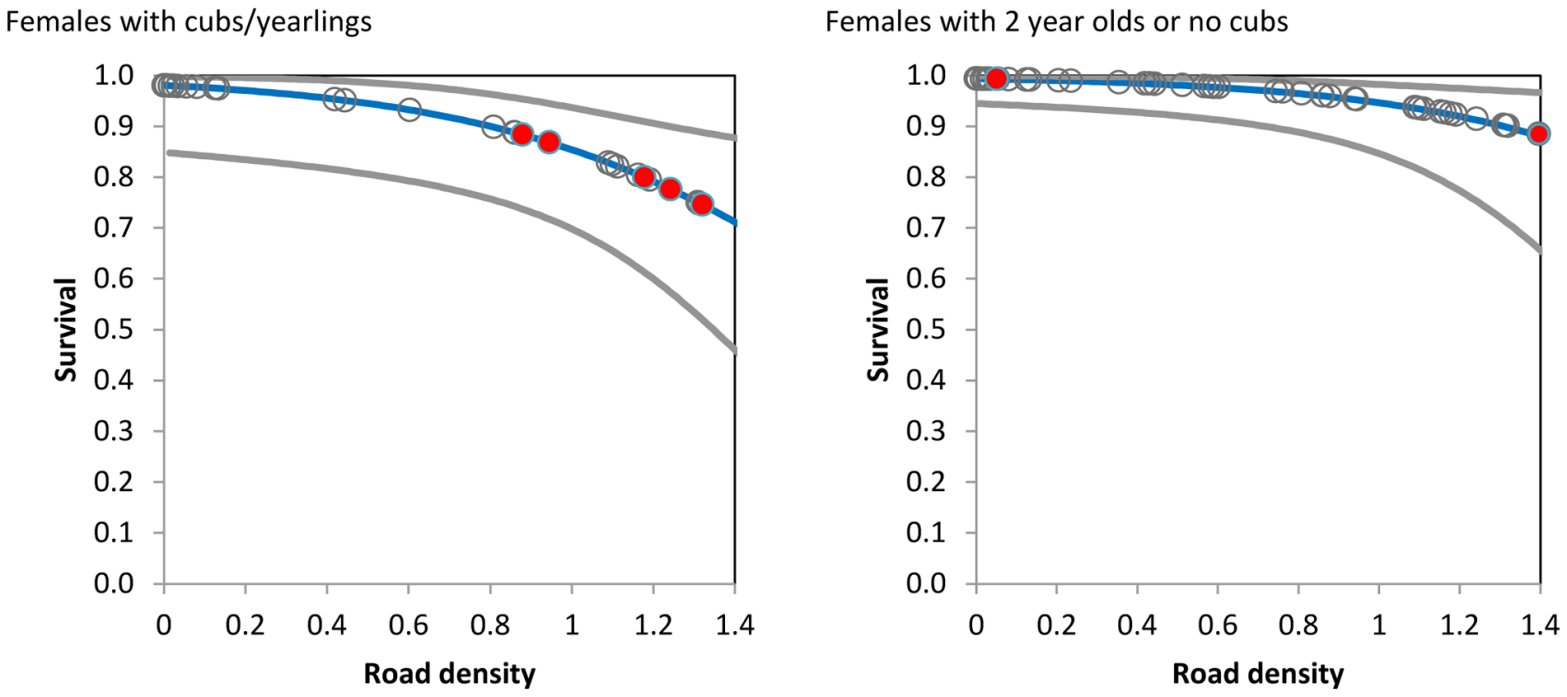
Estimates of survival of females as a function of road density and reproductive status (Model 1, **Table 3**) from the multi-state model analysis of grizzly bears in Alberta, Canada. Data points are given as grey circles with mortalities filled red. For the Females with 2 year olds or no cubs, there are 2 overlapping mortality points at a road density of 1.4. Grey lines indicate 95% confidence intervals for predictions.

The relationship between mortality risk and roads in model 1 suggests higher mortality risk as road density increases. Mortality risk was translated to survival (1-mortality risk) to allow comparison with the known fate estimates (Fig. 3). Females with cubs of the year or yearlings had lower survival rates at higher road densities compared to females without cubs or with two year olds. The distribution of road densities and associated fates showed that the majority of mortalities of females with cubs or yearlings occurred for bears in higher road densities. Survival rates of females with offspring were still higher than adult males (Fig. 2).

### Demographic model

The demographic model considered the effect of road density on adult and subadult females from the known fate analysis (Fig. 2) as well as a scenario where dependant offspring further reduced female survival as suggested by the multi-state analysis (Fig. 3). Transitions for the multi-state model without a dead state were used for the model given that mortality of females was modelled separately from reproduction. Data for estimates of yearling survival was too sparse to allow reliable estimates. We therefore used estimates from the NCDE ecosystem (S = 0.68 SE = 0.13, CI =  0.26–0.89 [40] to populate the demographic model.

**Figure 4:**
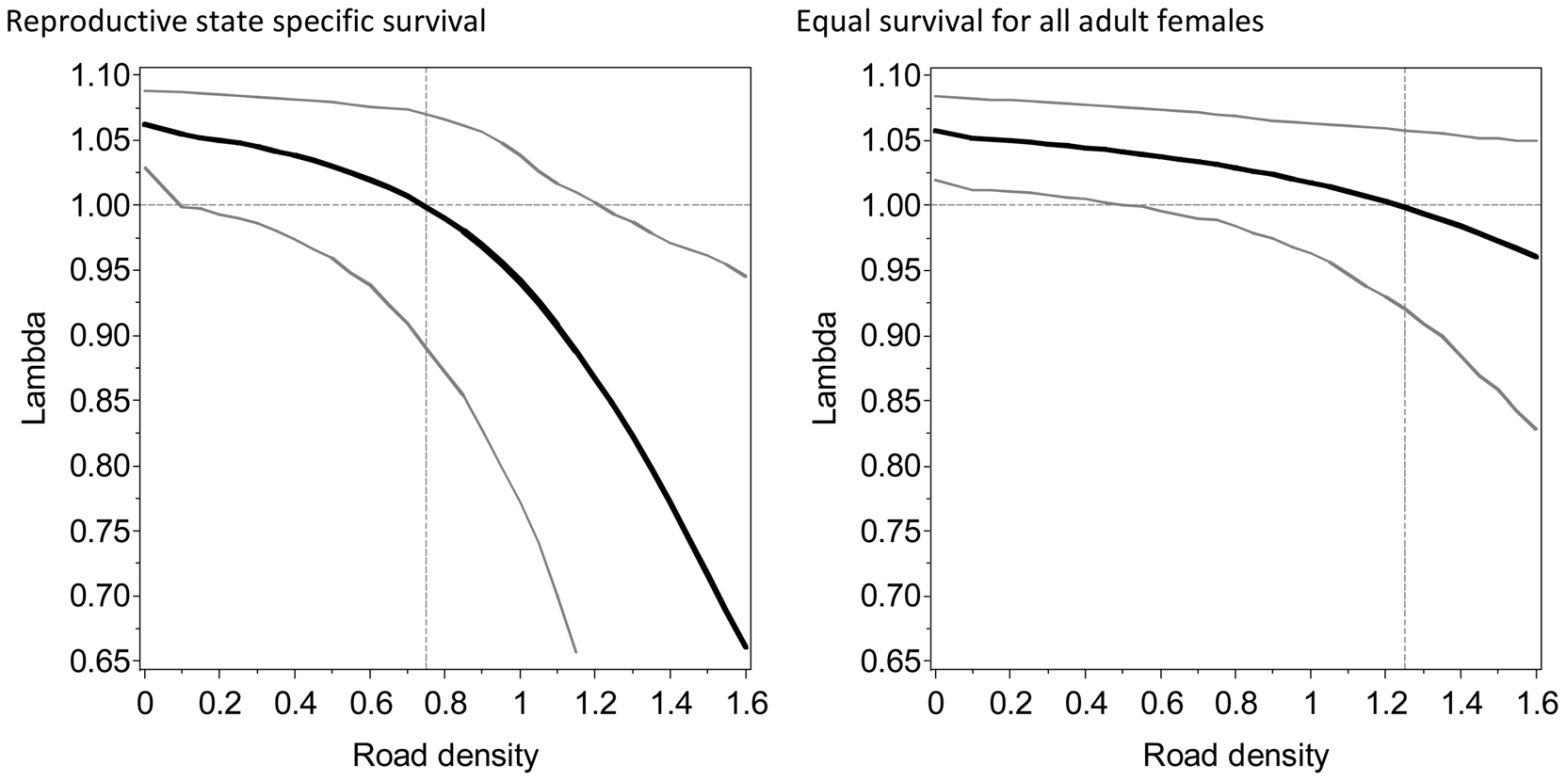
The effect of road density (km roads/km^2^) population trend (λ) assuming reproductive state specific survival (left, **Fig. 2**) and pooled adult female survival rates (right, **Fig. 2**) of grizzly bears in Alberta, Canada. Grey lines indicate 95% confidence intervals for predictions. The horizontal dashed line indicates population stability (λ = 1). The dashed vertical lines indicate threshold road densities where lambda = 1.

Results suggested that the threshold of road density where population rate of change became negative depended heavily on assumptions about the effect of road density on adult female survival (Fig. 4). If adult female survival was reduced for all adult females regardless of reproductive state as suggested by the known fate analysis then λ was reduced below 1 at adult and subadult female survival rates of 0.95 and 0.83 with corresponding road density of 1.25 (Fig. 2). If females with cubs/yearlings had reduced survival then λ was reduced to below 1 at road densities of 0.75 with corresponding survival of females with cubs/yearlings of 0.90 and females with 2 year olds or no cubs of 0.97 (Fig. 3) and subadult female survival of 0.93 (Fig. 2). Therefore, considering reproductive-state specific survival lowered the threshold of road density needed for a habitat to not be a sink habitat (where λ was less than 1).

**Figure 5:**
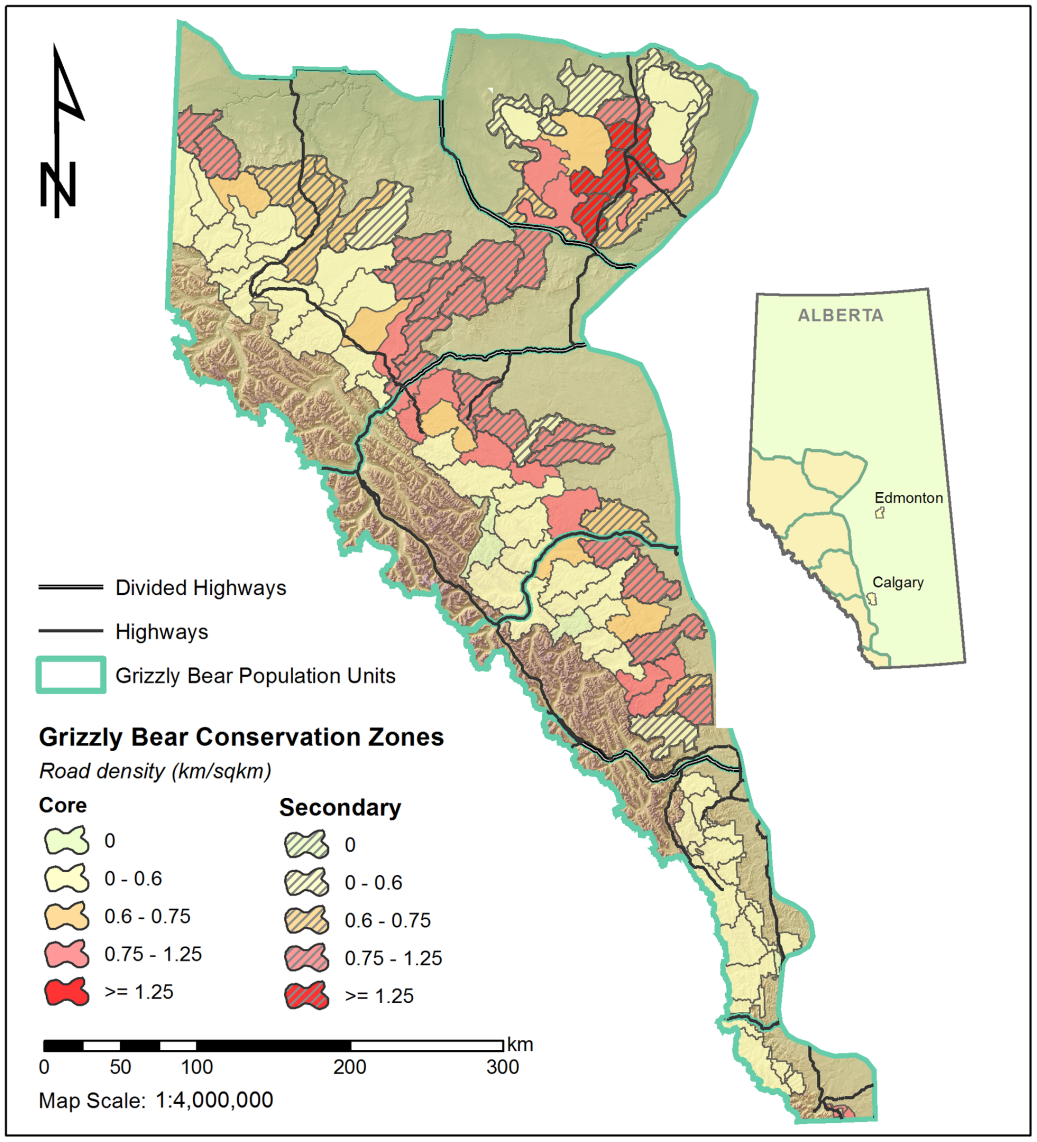
Road density for watershed units as subdivided by core and secondary areas for grizzly bear range in Alberta[17]. Mountainous areas (to the west of core secondary areas) were primarily protected parklands with low (<0.6 km roads/km^2^) road densities.

If projected spatially, the results of the demographic model can be used to illustrate source and sink watershed areas based on survival rates and predicted lambda (Fig. 5). Of watershed areas considered that in core areas (37,283 km^2^ total area), 82%, 18%, and 0% were in areas with road densities of less than 0.75, between 0.75 and 1.25, and >1.25 respectively. For secondary areas (24,779 km^2^ total area), 43%, 48%, and 9% were in areas with road densities of less than 0.75, between 0.75 and 1.25, and >1.25 respectively.

## Discussion

This analysis demonstrates that road density affects both the direct demography and trend of bear populations but introduces additional risk into reproduction and recruitment. Previous analyses [15] of bears in Yellowstone National Park and the surrounding area also concluded that human development was the principal factor influencing survival rates of grizzly bears. Based on previous demographic analyses it was suggested that sink habitats would be created [1] if adult female survival rate declined below 0.91. Our analyses suggested that the actual survival rate required for areas to not risk declining populations depends on reproductive state. If lower survival rates of females with dependent offspring is considered then the threshold of road density that bears can tolerate is reduced further (Fig. 4). The sensitivity or results to adult female survival rates and reproductive state follows from other demographic studies that demonstrate the highest sensitivity of population trend to adult female survival rates [44].

Our results illustrate that larger watershed areas outside of the mountainous zones have potential to have lower chance of population increase or stability if mortality risk near roads is not managed. This distribution of watersheds suggests that the majority of core areas are in areas of lower road density and therefore have the potential to be source habitats. Alternatively, 57% of secondary habitat are either in moderate (0.75–1.25) or high road density (>1.25) suggesting that these areas will require more intensive management to aid in population recovery and conservation actions. Currently the Alberta government is attempting to manage identified core and secondary conservation zones within each BMA at road densities of.6 km/km^2^ and 1.2 km/km^2^ respectively [17].

The multi-state model approach to estimation of reproductive rate was useful for the Alberta data set given that in most cases females were monitored for less than a complete interbirth interval. Therefore, potential bias could have occurred due to short monitoring intervals as well as potential capture related biases [39]. One factor that affects the estimation of reproductive rate is inclusion of three year old bears under the assumption that female bears in this age class can produce cubs therefore transitioning to the cub state when they are aged 4. Of the 16 three year olds monitored in this study, one gave birth to cubs at aged 4 which further justify inclusion of the aged 3 bears. Sample sizes precluded detailed age-specific estimation of reproductive rates in this analysis.

### Analysis assumptions and limitations

Our analysis assumes that overall road density in areas traversed by bears is indicative of the relative level of mortality risk. The effects of fragmentation of populations due to roads are not considered. Previous research suggests that gravel resource roads do not substantially fragment grizzly bear populations given the relatively low traffic level of these roads and lack of accompanying settlement associated with these roads [9], [49], [50]. However, this research has suggested that larger paved highways usually associated with settlement that bisect parts of Alberta do lead to fragmentation effects and in limited cases high mortality around gravel roads may cause fragmentation effects. Our analysis did not consider traffic volume on resource roads as an additional covariate for survival rates due to the challenges of monitoring traffic volumes across the wide expanse of grizzly bear habitat within the wide expanse of Alberta. However, it is likely that traffic volume will influence the relative degree of mortality risk and behaviour relative to roads [7], [14]. Subsequently, we suggest reduction and monitoring of traffic volume as a method to reduce and study the effects of mortality risk associated with resource roads.

In its current form, the demographic model does not consider possible increases in reproductive rate due to habitat quality increase caused by anthropogenic habitat. In general, covariates such as regeneration and canopy closure were not well supported in the MS model (i.e. was transition from N-C influenced by habitat). However we suspect this may have been due to limited power of the multi-state model to detect environmental relationships given the large number of parameters estimated relative to the sample size of the data set. For example, other studies [8] have shown that bears in regeneration habitat are more likely to increase their body condition but also have higher rates of mortality due to higher road densities in regeneration habitat.

Our estimates of lambda for watershed areas (Fig. 5) are restrictive in that it is assumed that emigration and immigration rates will be similar for each area so that estimates of trend will primarily be based upon reproduction and survival rates [51]. This is a simplification which is probably less valid at the relatively small scale of a watershed but more likely to be valid at the population unit scale. A study that used genetic and collar data determined that population units were defined by the larger highways that bisect the main grizzly bear management areas in Alberta (Fig. 1). Given the difference in scales, we suggest that estimates of lambda are best considered in relative rather than absolute terms. For example, areas with lambda values less than 1 should be considered as targets for restriction of access to roads to help restore viability of local grizzly bear populations. This general approach has been suggested by other studies [17], [52], [53], but the general methodologies have been based primarily on habitat modelling and not direct demographic modelling.

### Mechanisms for reduced survival of females with dependant offspring

The actual mechanism for lower survival rate of females with cubs and yearlings is unknown. Other studies [4] documented that females with cubs occurred closer to roads compared with other age and sex classes which could have been due to forage availability but also avoidance of males which displayed the reverse pattern. Therefore, it could be speculated that females with cubs are closer to roads and therefore will have higher risk than females without cubs or older cubs. Other factors, such as higher rates of encounter of females with cubs with males will also increase mortality risk due to females defending cubs against potential infanticide [54]–[56]. Regardless, our results suggest that mortality risk for bears is higher near roads and therefore if females with cubs or yearling occur closer to roads it is likely that they will have higher mortality rates.

### Comparison with other studies

The estimate of reproductive rate of 0.272 (CI = 0.20–0.41) for the this study is lower than the reproductive rates of 0.376 cubs per year (CI = 0.273–0.461) for the Northern Continental Divide Ecosystem [40] and 0.362 for Yellowstone National Park [1] but similar to the East Slopes study in Alberta [57]. The main reason for the reduction of the Alberta rate is lower litter size (2.11 compared to 2.28 for NCDE) and a lower stable proportion of females with cubs (25.8% compared to 32.2% for NCDE). Population rate of change (λ) was estimated a value of 1.04 (CI = 0.99–1.09) for Banff National Park [57] which borders the Yellowhead BMA. In the context of our study, this area would primarily have lower road densities and therefore this level of λ would certainly be possible (Fig. 4). A study of bear populations in the Northern Continental Divide Ecosystem of Montana [40], just south of the Alberta, also estimated λ at 1.036 (CI = 0.23–1.10) which was presumably due to the relatively high reproductive rate and relatively high rates of female survival.

## Conclusions

Previous analyses defined core and recovery zones in Alberta based upon a combination of resource selection function models scores (RSF) and road density thresholds of 0.6 and 1.2 for core and secondary habitats respectively [17]. [17]. The actual selection of these road density thresholds was based upon earlier studies of habitat selection relative to roads [16] and survival analyses (Boulanger and Stenhouse, *unpublished data*) that identified a linkage between road density and survival. Results from our demographic analysis suggest similar zones. In the context of our analysis, core grizzly bear conservation areas should allow survival rates of females with dependant offspring ishigh enough toensure an increasing population. Our demographic model estimates a threshold of at least 0.75 or lower when higher vulnerability of females with cubs relative to roads is assumed (Fig. 4). If similar survival rates for females and females with cubs are assumed then a road threshold of 1.25 is needed to ensure a stable population. We suggest that the 0.75 road density threshold is most applicable to ensure viable grizzly bear populations. However we also want to point out that working towards road densities that are lower than this threshold is a preferred conservation strategy and that the influence of human behaviour on roads plays a role in grizzly bear survival rates and population demographics. Further population inventory work to establish population status and the spatial distribution of grizzly bears can be coupled with measures of landscape metrics (roads and other variables) [58] and relative road use to evaluate recovery targets and land management decisions with testable hypothesis resulting from the findings of this work.

Because sampling was mainly centered in the Yellowhead and Grande Cache BMA's it is not possible to estimate a mean population trend for Alberta from our data set. In fact, the relationship between road density and lambda suggested in the demographic analysis demonstrates that there is a large degree of spatial variation in population trend (Fig. 5). This variation, and the sample size requirements needed to estimate a precise population trend [48] make it challenging to estimate λ in which the confidence limits would not overlap 1. In addition, this result demonstrates the challenges of obtaining a representative sample of collared bears across the landscape to allow unbiased estimates of mean λ especially since capture efforts often occur in roaded areas. We suggest that use of demographic models with covariates is a useful method to understand mechanisms of population trend, assess spatial variation in trend, and apply management guidelines to mitigate potential hazards for grizzly bears and other threatened species.

## Supporting Information

S1 Fig Map of mortality locations for bears used in the analysis. This map does not include locations of mortalities of bears that did not have radio collars or sufficient collar locations and associated road densities to allow inclusion in the analysis.
(TIF)

S1 Table Summary of grizzly bear mortalities included in the analyses.
(DOCX)

S2 Table Summary of data used in multi-state reproductive rate and survival analysis.
(DOCX)
