# Supplementary figures and images for: The Impact of Roads on the Demography of Grizzly Bears in Alberta
Source: PLoS One. 2014 Dec 22;9(12):e115535. doi: 10.1371/journal.pone.0115535 (PMC4274100; doi:10.1371/journal.pone.0115535)

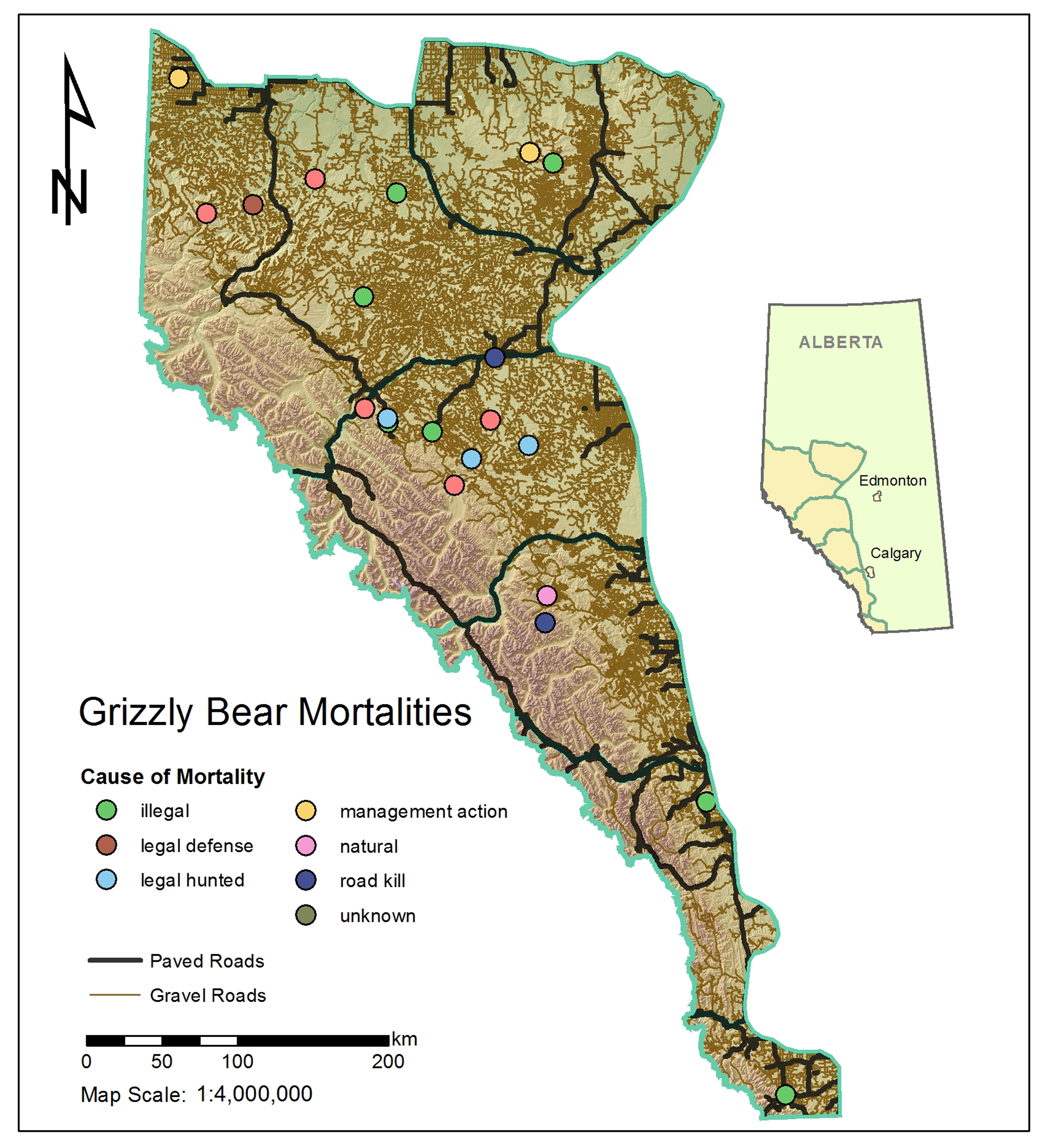

Supplement: S1 Fig — Map of mortality locations for bears used in the analysis. This map does not include locations of mortalities of bears that did not have radio collars or sufficient collar locations and associated road densities to allow inclusion in the analysis. (TIF) [file pone.0115535.s001.tif]
