# Supplementary material for: The Impact of Roads on the Demography of Grizzly Bears in Alberta
Source: PLoS One. 2014 Dec 22;9(12):e115535. doi: 10.1371/journal.pone.0115535 (PMC4274100; doi:10.1371/journal.pone.0115535)
Supplement: S1 Table — Summary of grizzly bear mortalities included in the analyses. (DOCX) [file pone.0115535.s002.docx]

Table S1: Summary of grizzly bear mortalities included in the analyses

| **Bear ID** | **Sex** | **Age class** | **Year** | **cause** | **Human cause** | **Distance to road (m)** | **Road type** |
| --- | --- | --- | --- | --- | --- | --- | --- |
| G020 | Female | Adult | 2002 | illegal | yes | 60 | primary |
| G026 | Female | Adult | 2000 | unknown | no | 25 | secondary |
| G027 | Female | Subadult | 2005 | legal hunted | yes | 340 | secondary |
| G036 | Female | Adult | 2002 | legal hunted | yes | 10 | secondary |
| G057 | Female | Subadult | 2003 | found dead | yes | 2 | secondary |
| G061 | Female | Subadult | 2010 | illegal | yes | 18 | secondary |
| G074 | Female | Subadult | 2003 | unknown | no | 9700 | NA |
| G204 | Female | Subadult | 2008 | illegal | yes | 70 | secondary |
| G223 | Female | Adult | 2009 | road kill | yes | 400 | secondary |
| G253 | Female | Adult | 2008 | unknown | uncertain | 51 | secondary |
| G024 | Male | Adult | 2001 | unknown | uncertain | 220 | secondary |
| G050 | Male | Adult | 2003 | legal hunted | yes | 4500 | secondary |
| G054 | Male | Subadult | 2002 | road kill | yes | 350 | secondary |
| G072 | Male | Subadult | 2004 | legal defense | yes | 1070 | secondary |
| G076 | Male | Subadult | 2004 | management action | yes | 0 | secondary |
| G088 | Male | Subadult | 2004 | illegal | yes | 250 | secondary |
| G201 | Male | Subadult | 2005 | natural | no | 1350 | NA |
| G207 | Male | Adult | 2005 | illegal | yes | 45 | secondary |
| G208 | Male | Adult | 2005 | management action | yes | 30 | secondary |
| G216 | Male | Adult | 2005 | illegal | yes | 66 | secondary |
| G227 | Male | Subadult | 2005 | illegal | yes | 437 | secondary |
| G267 | Male | Subadult | 2008 | management action | yes | 450 | secondary |
