# Supplementary material for: The Impact of Roads on the Demography of Grizzly Bears in Alberta
Source: PLoS One. 2014 Dec 22;9(12):e115535. doi: 10.1371/journal.pone.0115535 (PMC4274100; doi:10.1371/journal.pone.0115535)
Supplement: S2 Table — Summary of data used in multi-state reproductive rate and survival analysis. (DOCX) [file pone.0115535.s003.docx]

Table S2: Summary of data used in multi-state reproductive rate and survival analysis

| Bear | Age | years | Years in each state | | |  |  | Mortality cause |
| --- | --- | --- | --- | --- | --- | --- | --- | --- |
|  | mean | monitored | No  offspring | Cubs | Yearling | Two yr. olds | Dead |  |
| G002 | 18.5 | 7 | 4 | 1 | 1 | 1 | 0 |  |
| G003 | 7.8 | 8 | 5 | 1 | 1 | 1 | 0 |  |
| G004 | 10.5 | 10 | 6 | 2 | 1 | 1 | 0 |  |
| G007 | 5.0 | 8 | 5 | 2 | 1 | 0 | 0 |  |
| G010 | 15.5 | 10 | 6 | 2 | 1 | 1 | 0 |  |
| G012 | 7.0 | 8 | 5 | 1 | 1 | 1 | 0 |  |
| G016 | 10.6 | 8 | 5 | 1 | 2 | 0 | 0 |  |
| G020 | 5.5 | 6 | 4 | 1 | 1 | 0 | 1 | illegal |
| G023 | 16.8 | 18 | 9 | 3 | 3 | 3 | 0 |  |
| G026 | 3.0 | 1 | 1 | 0 | 0 | 0 | 1 | unknown |
| G027 | 12.5 | 7 | 4 | 1 | 1 | 1 | 1 | legal hunted |
| G028 | 7.5 | 6 | 4 | 1 | 1 | 0 | 0 |  |
| G034 | 21.5 | 3 | 2 | 1 | 0 | 0 | 0 |  |
| G035 | 3.5 | 2 | 2 | 0 | 0 | 0 | 0 |  |
| G036 | 3.5 | 3 | 2 | 1 | 0 | 0 | 1 | legal hunted |
| G037 | 8.1 | 9 | 7 | 1 | 0 | 1 | 0 |  |
| G038 | 15.5 | 3 | 2 | 1 | 0 | 0 | 0 |  |
| G040 | 5.0 | 6 | 4 | 1 | 1 | 0 | 0 |  |
| G042 | 18.5 | 3 | 2 | 0 | 0 | 1 | 0 |  |
| G048 | 4.0 | 3 | 3 | 0 | 0 | 0 | 0 |  |
| G060 | 20.5 | 2 | 2 | 0 | 0 | 0 | 0 |  |
| G061 | 9.0 | 2 | 1 | 0 | 1 | 0 | 1 | illegal |
| G064 | 20.5 | 3 | 2 | 0 | 0 | 1 | 0 |  |
| G065 | 6.0 | 3 | 3 | 0 | 0 | 0 | 0 |  |
| G070 | 5.5 | 3 | 2 | 1 | 0 | 0 | 0 |  |
| G071 | 9.0 | 5 | 3 | 1 | 1 | 0 | 0 |  |
| G073 | 5.0 | 4 | 3 | 1 | 0 | 0 | 0 |  |
| G074 | 5.0 | 1 | 1 | 0 | 0 | 0 | 1 | unknown |
| G075F | 5.5 | 3 | 2 | 1 | 0 | 0 | 0 |  |
| G077 | 4.7 | 4 | 3 | 1 | 0 | 0 | 0 |  |
| G086 | 8.5 | 2 | 2 | 0 | 0 | 0 | 0 |  |
| G091 | 15.5 | 2 | 2 | 0 | 0 | 0 | 0 |  |
| G092 | 7.5 | 4 | 2 | 1 | 1 | 0 | 0 |  |
| G093 | 8.5 | 2 | 2 | 0 | 0 | 0 | 0 |  |
| G095 | 7.5 | 2 | 2 | 0 | 0 | 0 | 0 |  |
| G096 | 4.5 | 2 | 2 | 0 | 0 | 0 | 0 |  |
| G111 | 6.3 | 6 | 4 | 1 | 1 | 0 | 0 |  |
| G113 | 4.5 | 2 | 2 | 0 | 0 | 0 | 0 |  |
| G117 | 7.5 | 2 | 2 | 0 | 0 | 0 | 0 |  |
| G118 | 5.0 | 4 | 3 | 1 | 0 | 0 | 0 |  |
| G119 | 4.5 | 2 | 2 | 0 | 0 | 0 | 0 |  |
| G204 | 4.0 | 4 | 3 | 1 | 0 | 0 | 1 | illegal |
| G205 | 12.5 | 2 | 2 | 0 | 0 | 0 | 0 |  |
| G223 | 21.0 | 6 | 3 | 1 | 1 | 1 | 1 | road kill |
| G224 | 5.5 | 3 | 2 | 1 | 0 | 0 | 0 |  |
| G225 | 3.5 | 2 | 2 | 0 | 0 | 0 | 0 |  |
| G238 | 5.0 | 6 | 5 | 1 | 0 | 0 | 0 |  |
| G251 | 4.5 | 4 | 4 | 0 | 0 | 0 | 0 |  |
| G253 | 3.0 | 1 | 1 | 0 | 0 | 0 | 1 | unknown |
| G254 | 9.0 | 8 | 5 | 1 | 1 | 1 | 0 |  |
| G258 | 6.0 | 3 | 3 | 0 | 0 | 0 | 0 |  |
| G260 | 7.5 | 10 | 6 | 3 | 1 | 0 | 0 |  |
| G265 | 6.5 | 2 | 2 | 0 | 0 | 0 | 0 |  |
| G269 | 16.5 | 3 | 2 | 0 | 1 | 0 | 0 |  |
